# On the relationship between silver concentration, surface roughness and photocatalytic activity of spin-coated TiO_2_ films

**DOI:** 10.1039/d6qi01631f

**Published:** 2026-08-10

**Authors:** Samah H. Alsidran, David J. Morgan, Philip R. Davies

**Affiliations:** a Cardiff Catalysis Institute, School of Chemistry, Cardiff University CF10 3AT UK daviespr@cardiff.ac.uk; b Department of Chemistry, College of Science and Arts, Najran University P. O. Box 1988 Najran 11001 Saudi Arabia; c HarwellXPS, Research Complex at Harwell, Rutherford Appleton Laboratory Harwell Science and Innovation Campus OX11 0FA UK

## Abstract

This study explores the impact of silver doping on the physical and photocatalytic properties of spin-coated TiO_2_ films. TiO_2_ films were loaded with varying amounts of Ag (0.1, 0.5, 1, 1.5, 2, and 5 wt%) synthesized *via* sol–gel spin coating and employed for the photocatalytic oxidation of stearic acid. The effects of spin speed and the number of deposited layers on film thickness and roughness were also examined. Increasing the spin speed from 500 to 7000 rpm reduced the film thickness by a factor of approximately 4.4, while spin coating 1 to 10 layers at 3000 rpm increased the thickness by a factor of *ca.* 6. Ag doping enhanced photocatalytic activity, with the 1.5 wt% Ag/TiO_2_ film exhibiting the highest efficiency, leaving approximately 2% stearic acid on the surface after 40 minutes of reaction. However, higher concentrations of silver (greater than 2 wt%) decreased activity, with 33% of stearic acid remaining on the surface for the 5 wt% Ag/TiO_2_ film. The 1.5 wt% Ag/TiO_2_ film displayed reduced activity when its surface roughness exceeded *ca.* 3 µm.

## Abstract 2

This study explores the impact of silver doping on the physical and photocatalytic properties of spin-coated TiO_2_ films.

## Graphical abstract

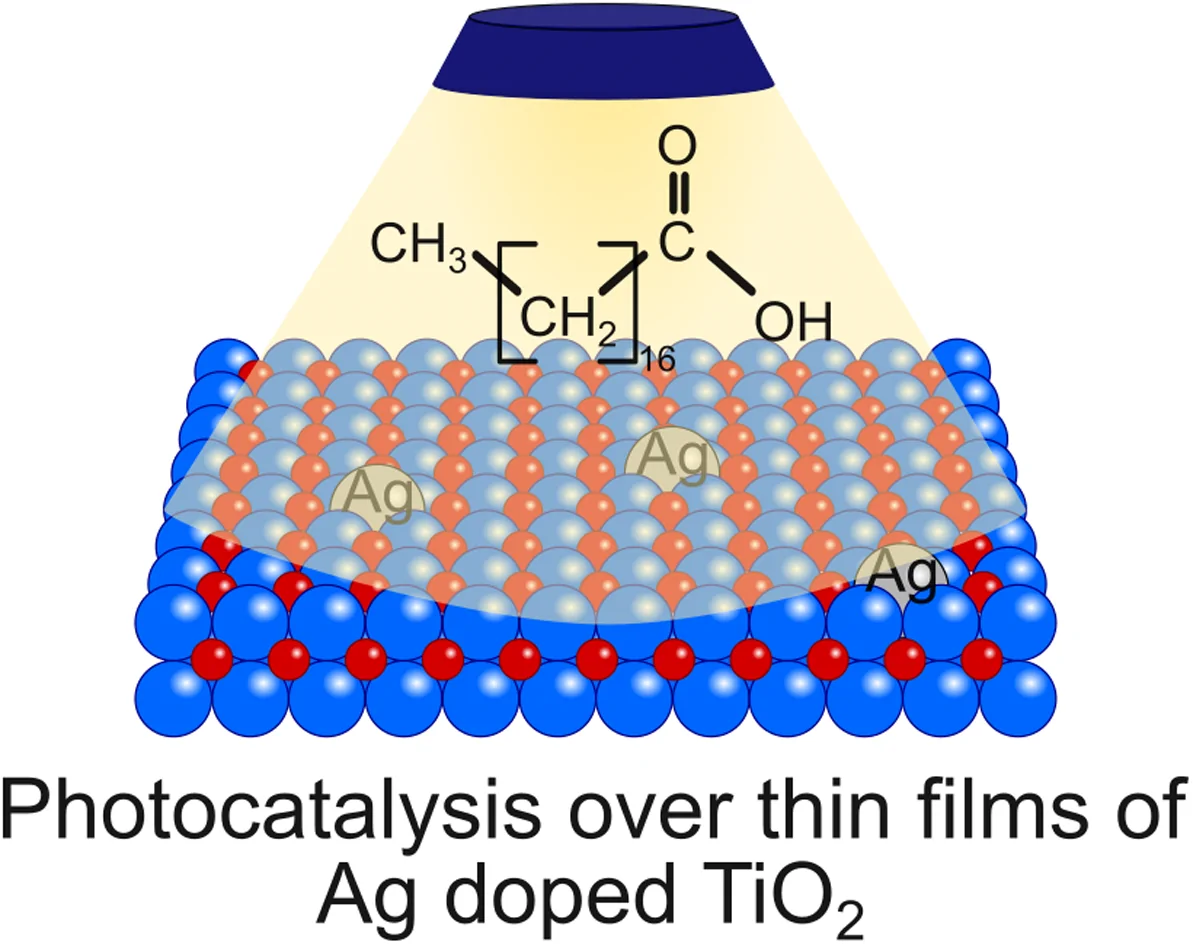

## 1. Introduction

Titanium dioxide-based self-cleaning glass windows that degrade organic pollutants at the surface through the photocatalytic and hydrophilic properties of the coating have attracted the interest of many glass manufacturers and is one of the areas in which Parkin has made important contributions.^1–6^ TiO_2_ is known as an ideal semiconductor for self-cleaning photocatalytic coatings and many other photocatalytic applications because of its high activity under UV light, affordability, low toxicity, high stability, and the simplicity of depositing it as a film.^7–10^ Furthermore, when exposed to UV light, TiO_2_ exhibits photo-induced hydrophilicity which can also contribute to a self-cleaning action.^11^ However, TiO_2_ coatings generally have two limitations: a relatively large band gap of around 3.2 eV, which limits the range of wavelengths from sunlight that it absorbs, and a fast recombination rate of the photogenerated electron–hole (e^−^/h^+^) pairs that outpace oxidation/reduction reactions at the surface and therefore limits its catalytic actvity.^12^ Numerous strategies have been attempted to overcome these restrictions and improve the photoactivity of titania, such as using a composite of TiO_2_ with a smaller band gap semiconductor and doping the titania lattice with a second element.^13–17^ Adding other ions to TiO_2_ has proven effective in increasing the photoactivity and is generally explained by the availability of mid-band gap energy levels, enabling the absorption of a wider range of wavelengths, or, in the case of metal nanoparticle formation, of trapping the excited electron increasing the lifetime of the electron–hole pair. However, added heteroatoms can also decrease catalytic activity; one explanation for these effects is that the foreign element acts as a recombination centre, but other effects are also possible, such as a change in the structure of the catalyst (*e.g.*, surface morphology) that influences charge transport or surface activity.^18^

Silver has been shown to be a promising dopant for TiO_2_,^16,19–23^ for example, Demirci *et al.*^24^ found increased activity with Ag concentrations below *ca.* 1 wt%, whereas Gogoi *et al.*^25^ reported the highest H_2_ production rate over 1.5% Ag/TiO_2_ catalyst. Bensouici *et al.*^26^ found that dip-coated TiO_2_ thin films with low concentrations of Ag (*ca.* 0.7 wt%) increased photodegradation, whereas films with high concentrations of Ag (*ca.* >3 wt%) were less active than pure TiO_2_. Some researchers attribute the reduced photoactivity at higher Ag contents to the phenomenon in which accumulated silver nanoparticles block light from reaching the active catalyst sites.^27,28^ On the other hand, some studies have shown the highest photoactivity in TiO_2_ films loaded with higher levels of silver, Bhardwaj *et al.*^29^ for example, reported an increase in activity for the TiO_2_ photocatalyst at silver concentrations of *ca.* 5 wt%, while Komaraiah *et al.*^30^ found enhanced activity of TiO_2_ film prepared with a maximum 7 wt% Ag. According to Jaafar *et al.*^31^ the improved performance at higher Ag loading can be attributed to an optimal distribution of metallic species and the maximum concentration of surface oxygen vacancies, which promote electron–hole pair separation. Given these contradictory results in the literature, it is important to continue to investigate the effect of Ag loading on the photocatalytic behaviour of TiO_2_.

Achieving efficient self-cleaning photocatalytic coatings requires a rapid, reproducible, and cost-effective method that produces transparent thin films with controllable properties such as thickness and roughness. These parameters are critical, as optimizing them can enhance photocatalytic activity by generating more charge carriers with sufficient lifetimes to reach the surface before recombination. Thin films can be prepared *via* sol–gel solutions, followed by spin-coating, dip-coating, or spray-coating.^14^ Among these approaches, spin-coating offers precise control over film thickness through adjustments in spin speed or by applying multiple layers of the precursor solution.

In this study, we investigate the effect of Ag doping on the photocatalytic activity of TiO_2_ films, specifically for degrading the C–H bond in stearic acid. We examine how variations in Ag loading and deposition parameters influence the hydrophilic properties of the coatings, film thickness, surface roughness, and overall photoactivity. Our findings reveal that even small amounts (<5 wt%) of Ag dopant promotes the photodegradation of stearic acid in the presence of ambient oxygen, producing only CO_2_ and H_2_O, with no detectable by-products on the photocatalyst surface. Importantly, the photocatalytic performance is strongly dependent on the deposition conditions. These results provide valuable insights for the design of more efficient self-cleaning and environmentally responsive surfaces.

## 2. Experimental

### 2.1 Film formation

Titanium(iv) isopropoxide (TTIP) (C_12_H_20_O_4_Ti, +98%, Thermo Fisher Scientific) and silver nitrate (AgNO_3_, Sigma Aldrich) were used as precursor, ethanol (Sigma Aldrich, anhydrous 99.8%) as a solvent, and glacial acetic acid as a stabilizer. All solvents and chemicals were of analytical grade and were used without additional purification. For a 1 wt% Ag/TiO_2_ catalyst, 3.052 mL of TTIP was hydrolyzed in 10 mL of ethanol and left to stir at room temperature. After 30 minutes of stirring, 1 mL of glacial acetic acid was added to the solution and left to stir until a homogenous solution of TiO_2_ was obtained. To prepare a 1 wt% Ag loaded coating, 1.295 × 10^−2^ g of silver nitrate was dissolved in ethanol and added to the TiO_2_ solution under magnetic stirring; after 30 min, 1 mL of glacial acetic acid was added to obtain a homogeneous solution. The same procedure, with appropriately scaled quantities of AgNO_3_ was used to prepare 0.1, 0.5, 1.5, 2, and 5 wt% Ag-loaded TiO_2_ catalysts.

For the experiments exploring the effects of surface roughness, 0.1 g of photocatalytically inert silica nanoparticles (SiO_2_, 99%, ∼0.5–10 μm, Honeywell Fluka) were added to 1.5 Ag wt% catalyst precursor solutions prior to spin-coating.

Glass slides were cut into *ca.* 2.5 × 2.5 cm squares, cleaned using an ultrasonic bath in water, ethanol, and acetone for 10 min each, and dried in an oven at 40 °C. The pure and Ag-loaded TiO_2_ solutions were spin-coated onto the clean glass substrates using a Laurell (Lansdale, PA, USA) WS-650MZ-23NPP system. A 100 μL solution was deposited onto a glass substrate at 3000 rpm for one minute. The samples were placed in the oven at 110 °C for 15 min to evaporate the solvent, after which the samples were calcined in the muffle furnace in air at 400 °C for 2 h with a heating rate of 5 °C min^−1^ and cooled to room temperature. Powder samples for examination by X-ray diffraction, were synthesised by drying a portion of the solutions used to prepare the spin coated catalysts in the oven overnight at 110 °C before calcining in air at 400 °C for 2 h.

### 2.2 Photocatalytic testing

The photoactivity of the coatings was studied by monitoring the area of the CH stretching peak of a thin layer of deposited stearic acid using attenuated total reflection Fourier Transform Infrared (ATR-FTIR) spectroscopy on a PerkinElmer (Waltham, MA, USA) Frontier spectrometer.^32^ A 100 µL of 0.05 mol L^−1^ stearic acid (Sigma Aldrich, reagent grade, 95%) in chloroform (reagent grade) was dispensed onto a spinning photocatalytic film at 2000 rpm for 30 s. The film was left to spin for a further 30 s to guarantee the complete evaporation of chloroform. A modified UVLED photocatalytic test reactor, established as part of the EU-funded PCATDES project, was used to illuminate the films in air. It provides a calibrated, adjustable light source with a maximum light intensity of 1.9 kW m^−2^ at a wavelength of 365 ± 2 nm at a distance of 0.1 m from the UV-LEDs.^33,34^ Consecutive ATR-FTIR spectra were collected before and after illumination until the reaction reached a specific time limit (40 min). The degraded percentage of stearic acid was evaluated from the area of the C–H absorption peaks (2917 and 2849 cm^−1^) after subtracting a constant background using OriginLab software 2023b. Each measurement reported below is the result of at least 3 separate experiments with the error representing the range of results.

### 2.3 Film characterization

#### 2.3.1 Varying film thickness by layering and spin speed

The 1.5 wt% Ag-loaded sample was selected to test the impact of film thickness and roughness. The 1.5 wt% Ag/TiO_2_ solution was spin-coated for one minute onto the glass substrate, and a range of spin speeds between 500 and 7000 rpm was applied to achieve different thicknesses. A total of one to ten layers of 1.5 wt% Ag film were produced on top of one another, and each layer was allowed to partially dry on the spin coater before the addition of the consecutive layer.

A Zeta-20/300 optical profiler (KLA Instruments) was used to measure film roughness. The roughness measurements were conducted by measuring the average difference between peaks and valleys (“*R*_q_”) from the 2D profiles of the films. The thicknesses of the coatings were measured at the edge of coatings or in experiments where a section of the glass substrate was covered using sticky tape and removed before the drying and calcination stages. This created a step between the uncoated glass and the photocatalytic coating which could be measured.

#### 2.3.2 Spectroscopic characterisations

XP spectra of the coatings were obtained with a ThermoFisher Scientific K-Alpha^+^ photoelectron spectrometer, using micro-focussed monochromatic Al Kα X-ray source with a photon energy of 1486.68 eV and operating at 72 W (6 mA × 12 kV). Spectra were collected using the 400-micron spot mode (which averages the signal from an elliptical area of *ca*. 600 × 400 microns) and pass-energies of 40 eV for high-resolution scans and 150 eV for survey scans with 0.1 eV and 1.0 eV step sizes respectively. CasaXPS (version 2.3.25) was used to analyze the data using a Shirley background and Scofield cross sections. Binding energies are calibrated to Ti 2p_3/2_ peak at 458.8 eV and have an accuracy of ±0.25 eV.^35^

Powder X-ray diffraction (XRD) data over the range 2*θ* = 10–80°, were obtained from powders synthesised in the same process as the films (see above) using a PANalytical X'Pert Pro diffractometer with a monochromatic Cu Kα source (*λ* = 0.154 nm) operated at 40 kV and 40 mA. DRS-UV spectroscopy was performed on the deposited films using an Agilent Technologies Cary Series UV-Vis Spectrometer scanning from 800 to 200 nm.

## 3. Results and discussion

### 3.1 The effect of Ag doping on the activity of anatase TiO_2_ films

**Fig. 1:**
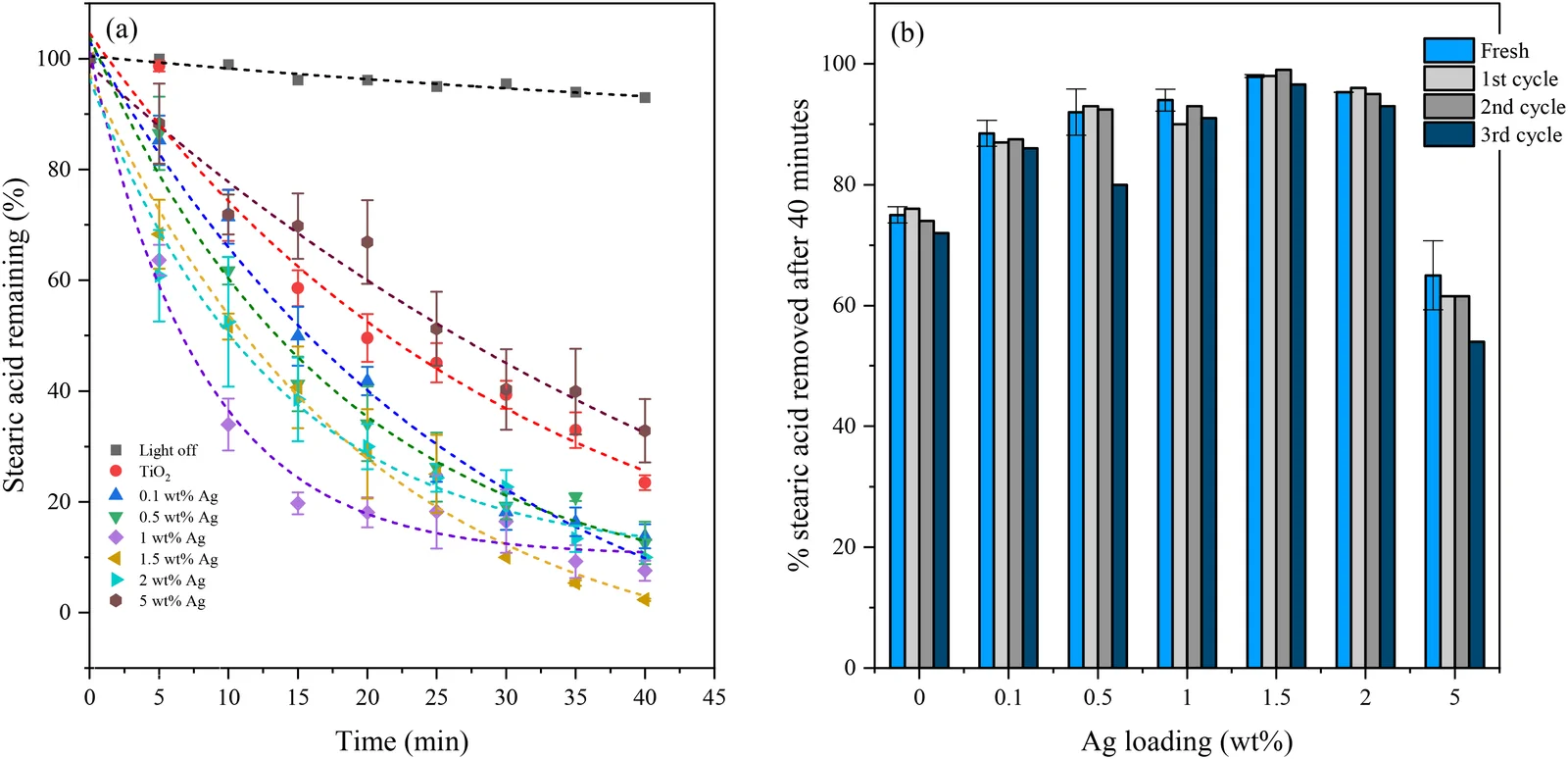
(a) Effect of silver loading on the photocatalytic performance of TiO_2_ spin-coated films; (b) The efficiency of the photocatalytic decomposition over 4 cycles represented by the % of stearic acid removed after exposure to 365 nm light for 40 minutes (% area errors are only shown for the fresh samples but were of similar magnitudes for all samples).

The impact of increasing silver loadings on the activity of spin-coated TiO_2_ films can be seen in Fig. 1(a) and (b). It is evident that incorporating Ag into TiO_2_ films at low concentrations enhances the photocatalytic activity with 1 and 1.5 wt% Ag film exhibiting the highest photoactivities. At silver concentrations above 1.5 wt% activity decreases with the 5 wt% Ag loaded sample showing a rate slightly lower than that of the pure TiO_2_ coating, consistent with some of the previous studies.^24–26,36^

For all the films, reusability is good, with activity remaining consistent for all of the samples for three coats of stearic acid and only decreasing by a few percent on the fourth coating, Fig. 1(b).

**Fig. 2:**
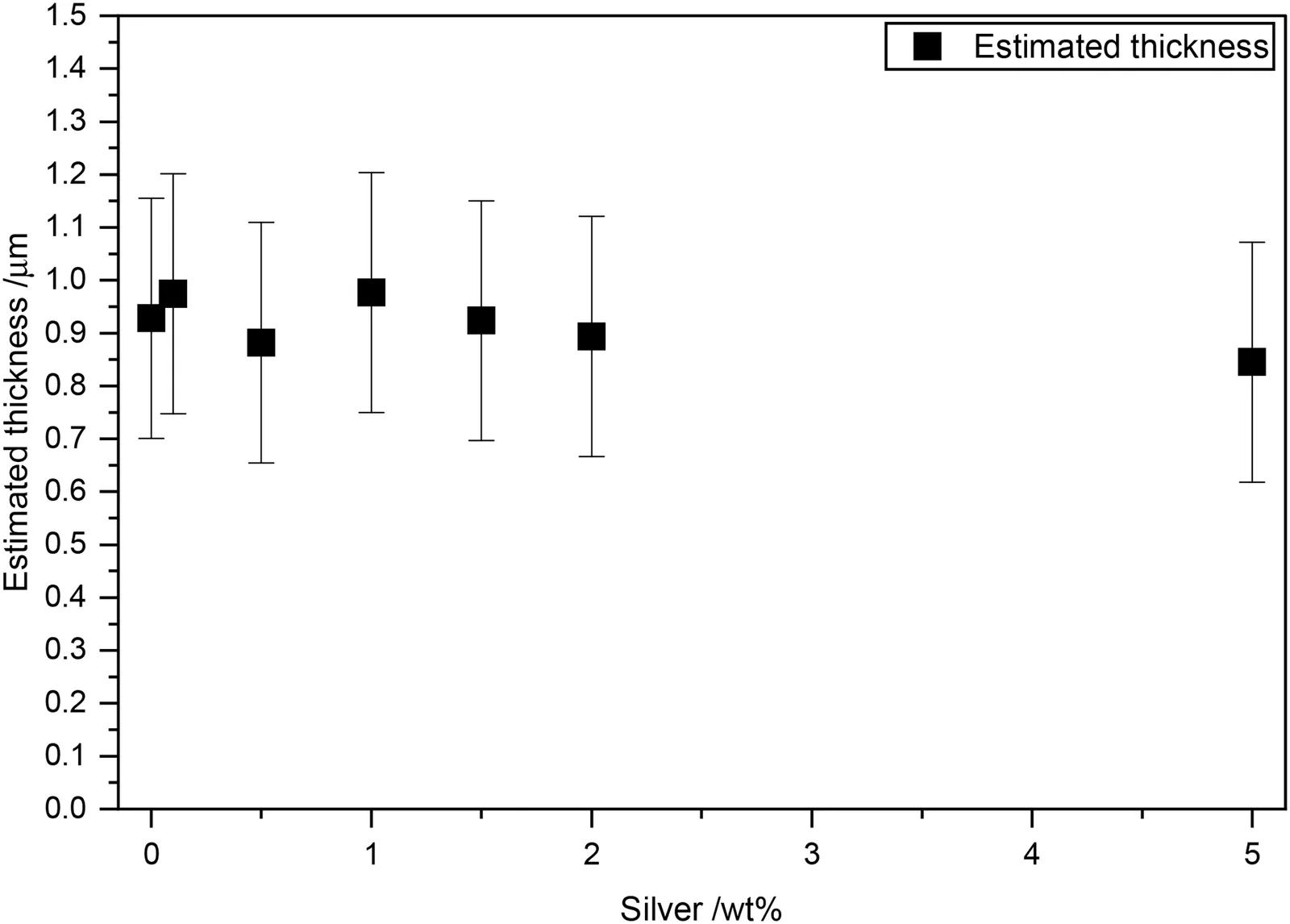
Estimated thickness of Ag loaded TiO_2_ films deposited at 3000 rpm. Thicknesses are estimated from optical profilometry data.

**Fig. 3:**
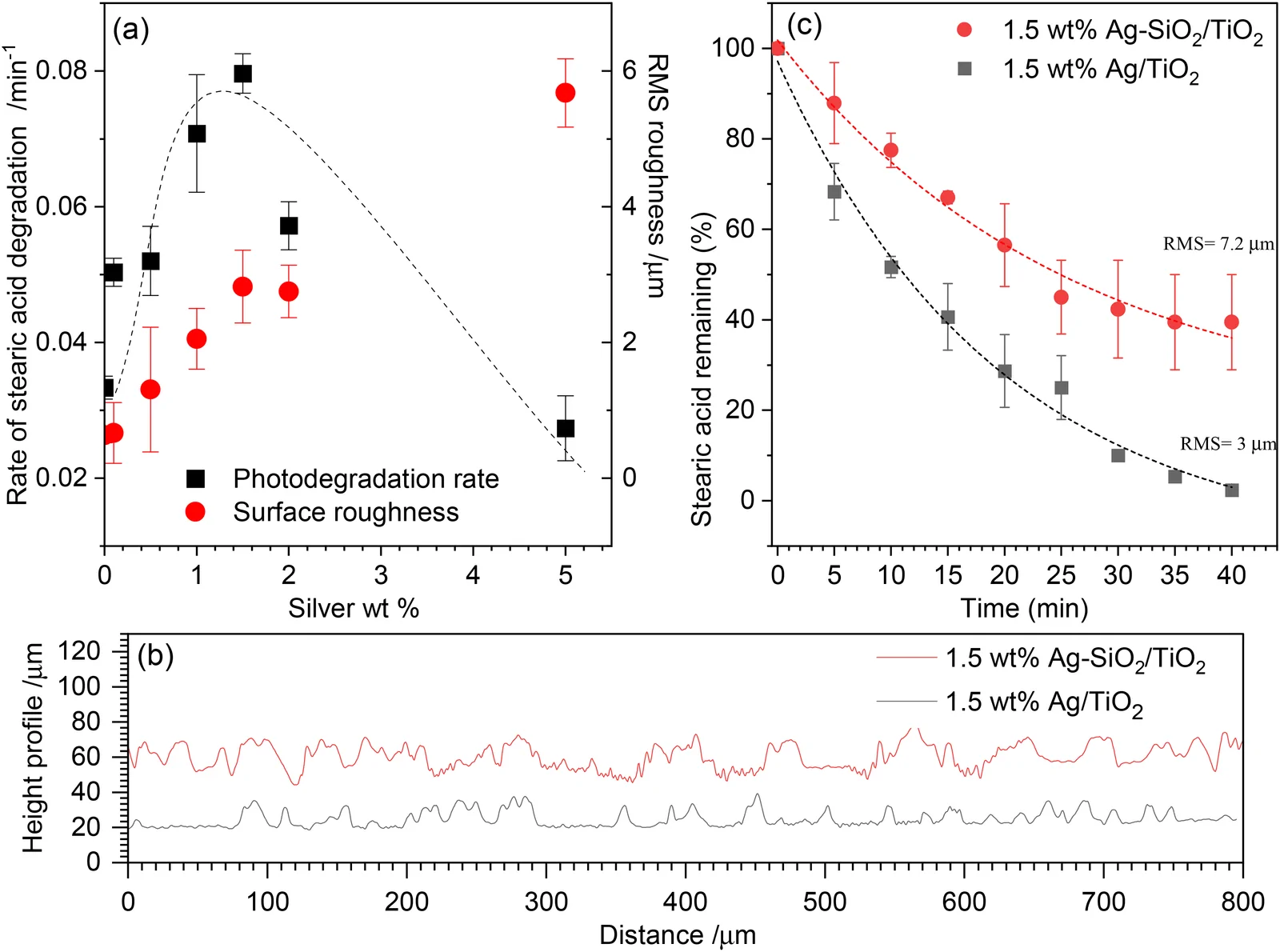
(a) Variation in surface roughness and photocatalytic degradation rate as a function of Ag wt% (dotted line added as a visual guide only); (b) Comparison of representative height profiles with and without the addition of silica nanoparticles; (c) Effect of silica nanoparticles on the 1.5 wt% Ag/TiO_2_ film roughness and photocatalytic activity.

In contrast to our previous study,^37^ which showed that Cu doping influenced film thickness and photoactivity, optical profile measurements demonstrate that despite varying Ag loading levels influencing activity, film thickness remains largely unaffected, Fig. 2. We have therefore investigated the effect of other critical film parameters on activity which could have an impact, in particular surface roughness as measured by the root mean square variation in topography on the films (*R*_q_).^38^ Fig. S1 demonstrates that increasing the Ag doping level leads to the formation of agglomerates on the film surface, resulting in an increase in RMS from around 0.63 microns for TiO_2_ only, to 2.8 microns for films with 2 wt% Ag, followed by a sharp rise to about 6 microns for the 5 wt% Ag-loaded film as shown in Fig. 3(a). It has been reported that increased surface roughness can improve activity by increasing the surface area.^39^ Lee *et al.*^40^ suggested that the surface roughness of the N-loaded TiO_*x*_ film could significantly influence photocatalysis. In contrast, Takeda *et al.*^41^ argued that surface roughness does not significantly affect the activity of the coatings. To separate out the effects of roughness and Ag concentration in this case, the roughness of the most active film (1.5 wt% Ag) was increased by adding photocatalytically inert silica nanoparticles to the precursor solution before deposition. This method raised the film roughness from 2.7 microns to 7.2 microns, Fig. 3(b). Fig. 3(c) shows that the increased roughness decreased activity, with only 40% of stearic acid decomposed after 40 min compared with almost complete degradation in the absence of the silica. This decrease may in part be attributed to the SiO_2_ particles blocking TiO_2_ sites, but at the same time the disruption of the TiO_2_ film by the silica can be expected to increase the overall surface area.

### 3.2 Characterization

XRD patterns of the samples, Fig. S2, show that the pure and Ag-loaded samples consist only of anatase TiO_2_. Our data show no peaks indication of silver, implying that the silver is either incorporated into the titanium dioxide lattice or it forms an amorphous solid. No significant variation in the band gap of the TiO_2_ films with silver concentrations was observed shown in Fig. S3 and tabulated in Table S1.

**Fig. 4:**
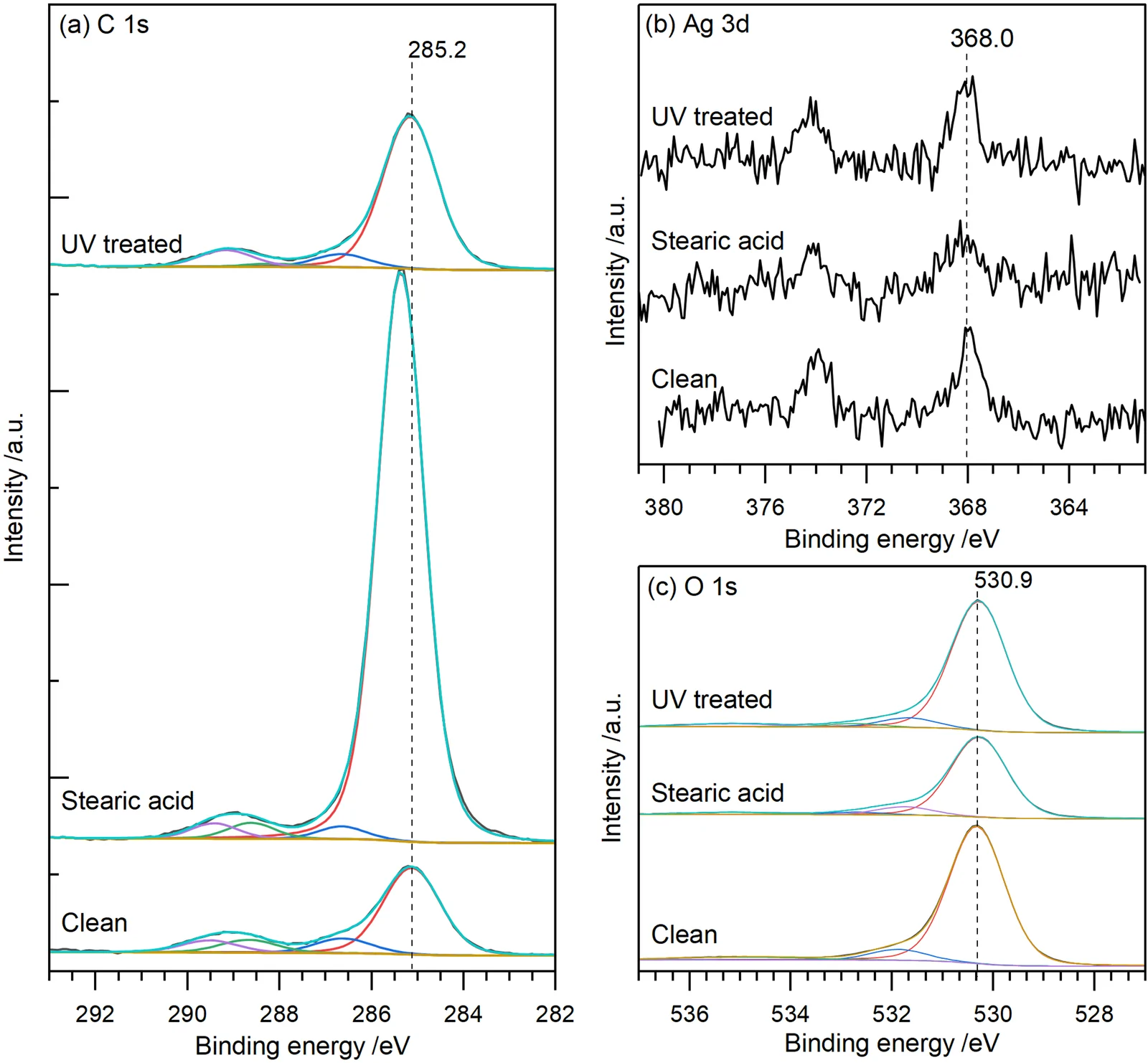
XP spectra of the C 1s, O 1s and Ag 3d regions, showing photocatalytic behaviour of 1.5 wt% Ag/TiO_2_ catalyst. (a) Fresh 1.5 wt% Ag/TiO_2_ spin coated at 3000 rpm. (b) 1.5 wt% Ag/TiO_2_ film with a layer of stearic acid spin coated on top. (c) 1.5 wt% Ag/TiO_2_ film with a layer of stearic acid spin coated on top after 40 min UV illumination.

XP spectra of the films, Fig. 4, show the Ag 3d_5/2_ and Ag 3d_3/2_ peaks at 368.0 eV and 374.2 eV respectively, indicative of an Ag(i) state, although the Ag binding energy is known to be a poor predictor of oxidation state. Increasing loadings of Ag result in higher intensities of the Ag 3d peaks but no other changes in peak positions or in the C 1s or O 1s spectra, Fig. S4. The deposition of stearic acid leads to the attenuation of the Ti 2p, O 1s and Ag 3d peaks but this attenuation disappears post UV treatment. In the O 1s region, the main peak at 530.3 eV corresponds to TiO_2_, whereas the component at ∼532 eV is attributed to the SiO_2_ substrate. The smaller component at ∼533 eV can be attributed to hydroxides or carbonates on the TiO_2_ surface. In the C (1s) spectra the components at 285.2, 286.6, 288.6, and 289.5 eV on the clean surface are attributed to C–C, C–O, C=O, and –COO, respectively of a small amount of surface contamination and some of the starting materials still trapped in the calcined films. The deposition of a stearic acid film results in an increase in the intensity of the main peak and a 0.2 eV shift to higher binding energy at 285.4 eV. After photocatalytic reaction the main peak shifts back to 285.2 eV and 88% of the carbon added in the stearic acid is lost from the surface.

Water contact angle measurements were performed on the samples both before and after 60 minutes of LED light exposure at 365 nm, as shown in Table S2. All pure and Ag-loaded TiO_2_ films displayed contact angles below 100°, indicating a hydrophilic character but the 5 wt% Ag/TiO_2_ sample demonstrated a contact angle of 7.4° indicating superhydrophilicity. Following UV irradiation, a general decrease in contact angles was observed across all samples, with the exception of the 5 wt% Ag-loaded film, which already exhibited super-hydrophilic properties prior to light exposure. This behaviour aligns with the well-established photo-induced hydrophilic transformation of TiO_2_ surfaces, where UV light generates surface hydroxyl groups and destroys organic contaminants.^42^ This hydrophilicity could be part of the reason for the poor photocatalytic activity of the 5 wt% Ag loaded sample since it would lead to a weaker interaction between the TiO_2_ film and the hydrocarbon chain of the stearic acid. Previous studies have suggested that the strength of interaction between the film and the adsorbate is related to the photocatalytic activity.^9,43^

### 3.3 The effect of spin coating parameters on the photoactivity of 1.5 wt% Ag/TiO_2_ film

**Fig. 5:**
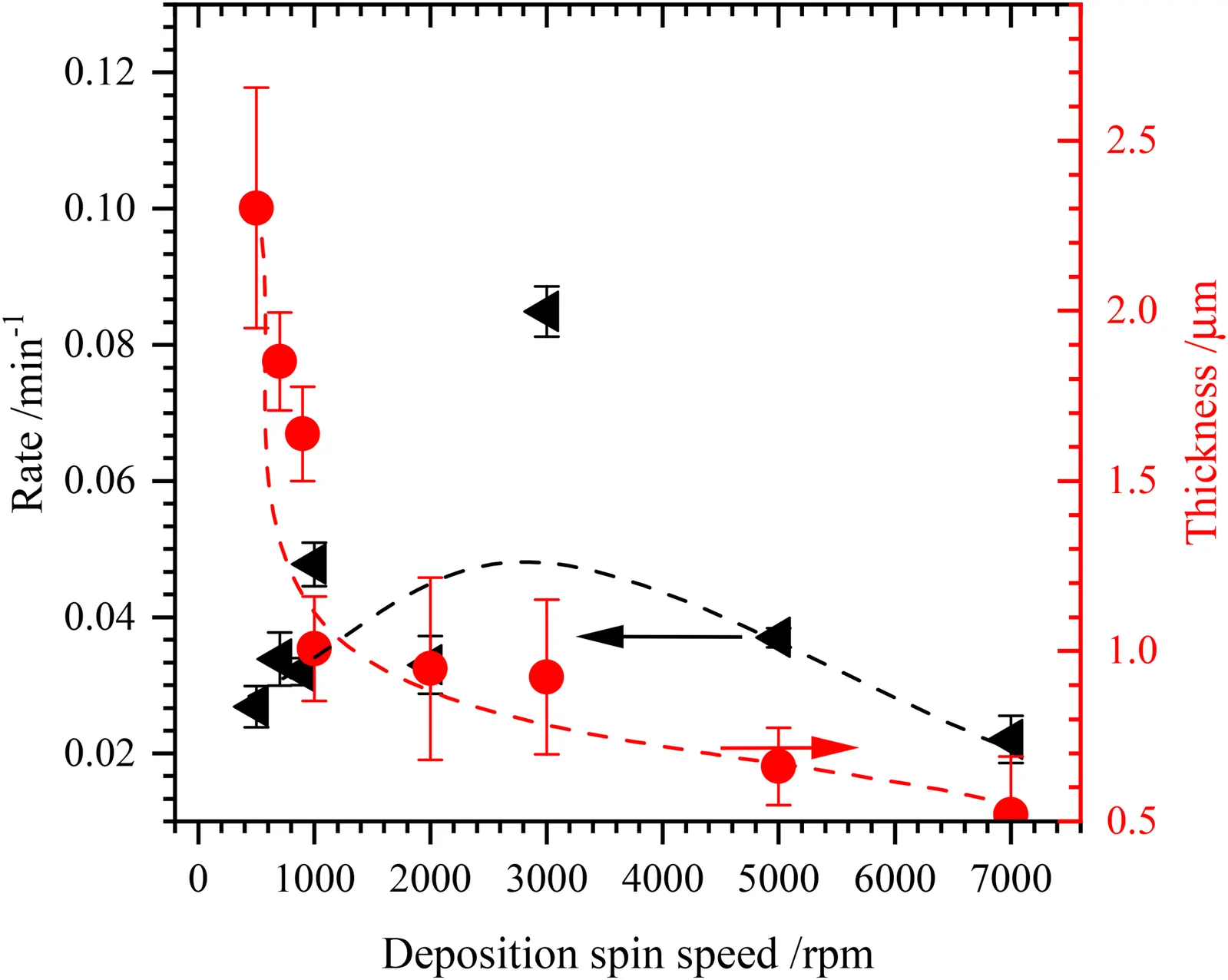
Reaction rates (black triangles plotted against left axis) and thicknesses (red circles plotted against the right axis) of 1.5 wt% Ag-loaded TiO_2_ film plotted against spin coating speed. Dotted lines to guide the eye.

The photocatalytic degradation of stearic acid was evaluated on a series of 1.5 wt% Ag/TiO_2_ films deposited at spin speeds ranging from 500 to 7000 rpm, and in Fig. 5 the activity and film thicknesses are compared against the different deposition spin speeds. Typically, higher spin speeds result in thinner films due to the increased centrifugal force acting on the liquid precursor. This higher force at elevated spin speeds spreads the precursor solution evenly on the substrate, producing thinner and more uniform films. In contrast, lower spin speeds generate thicker and rougher films, Fig. S5.^44^ The thickness decreases steadily from 2.3 to 0.5 µm with increasing spin speed from 500 to 7000 rpm. However, the data also show no consistent relationship between activity and spin speed/film thickness, except for the film spin-coated at 3000 rpm, which consistently shows a uniquely high activity. Since the films spin-coated at 3000 and 2000 rpm exhibit similar roughness and thickness values, the increased activity of the 3000 rpm film cannot be attributed to its thickness and at present we have no explanation for this unusual activity.

### 3.4 The effect of layering on the film thickness and photoactivity

**Fig. 6:**
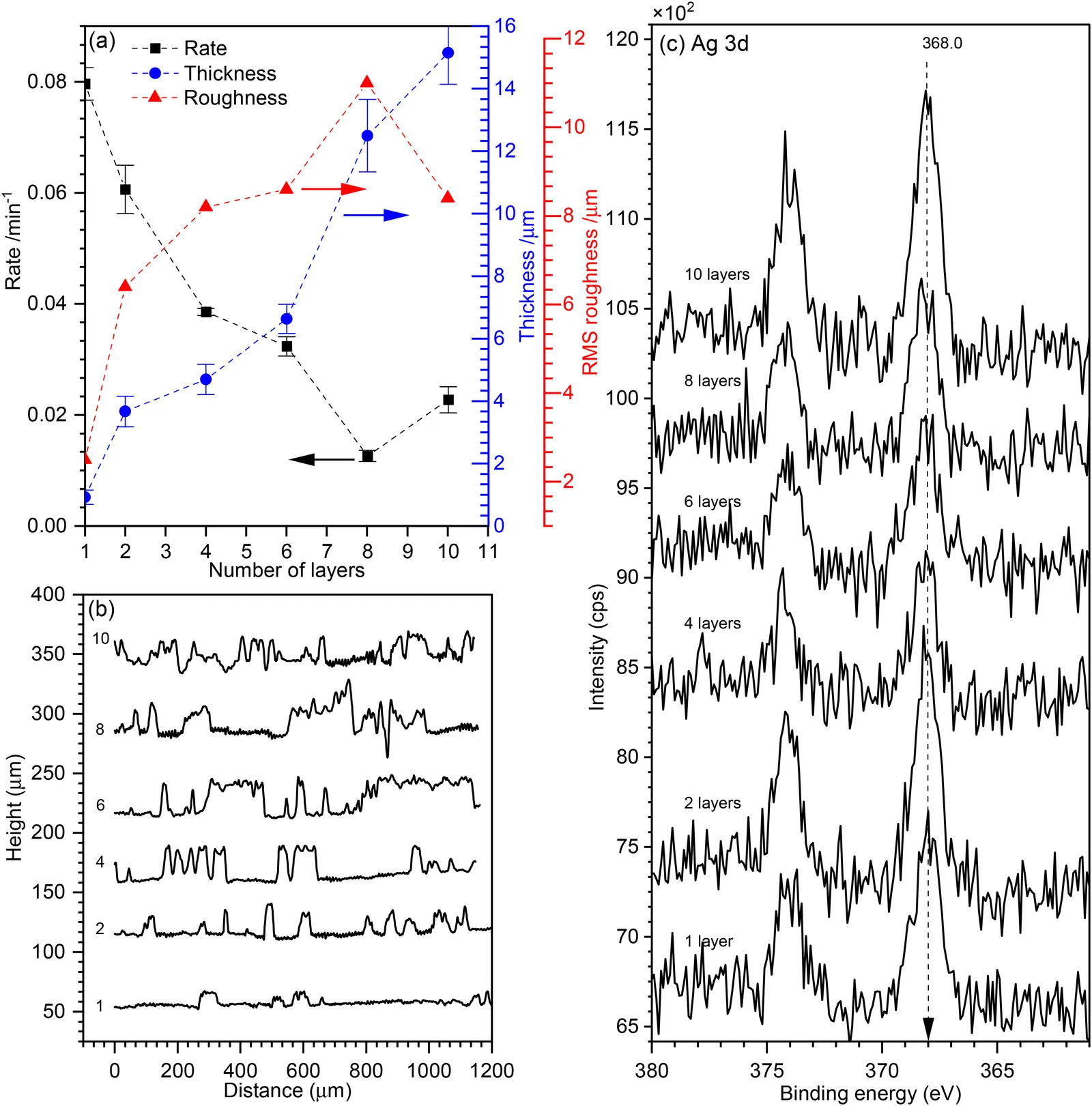
(a) Comparison of the effects of number of spin coated (3000 rpm) layers of 1.5 wt% Ag-loaded TiO_2_ on the photocatalytic degradation rate (left hand axis), and the roughness and thickness of the films (right hand axis); (b) 2D profiles of 1.5 wt% Ag loaded-TiO_2_ films showing the effect of multiple layers deposition on the surface roughness; (c) XP spectra of the Ag 3d region showing consistent silver concentration and oxidation state as the number of layers increases.

Successive deposition of 1.5 wt% Ag coatings is an alternative approach to increasing the thickness of the final coatings. Spin coated layers are dried but not calcined before another coat is applied on top. With the addition of a total of 10 layers, the film thickness increases from 0.92 µm to 15.1 µm and the roughness from 2.7 µm to 8.4 µm, Fig. 6(a) and (b). XP spectra of the Ag 3d region, Fig. 6(c), show that the concentration of silver at the surface remains constant as more layers are added and all the coatings have a Ag 3d_5/2_ binding energy of 368.0 eV, consistent with Ag(i). Plotting the calculated photodecomposition rate against measured film roughness, confirms a trend of decreased photocatalytic reaction rate for rougher films (RMS roughness > *ca.* 3 µm), which is consistent with the results in Fig. 3.

## 4. Conclusion

We have investigated the influence of silver concentration and film deposition parameters on the photocatalytic performance of anatase TiO_2_ films fabricated through a sol–gel spin-coating method. Our results demonstrate that TiO_2_ films loaded with silver concentrations ranging from 0.1 wt% to 2 wt% exhibit enhanced photocatalytic activity compared to pure TiO_2_ films. However, this activity decreases at higher silver loadings, and at 5 wt% Ag the activity is lower than that of pure TiO_2_. Band gap measurements show that this changing activity is not due to silver influencing the band gap. The photodecomposition of stearic acid over films with 1.5 wt% Ag was complete, leaving no remaining stearic acid on the surface. We investigated the 1.5 wt% Ag loaded coatings in more depth and found that the spin coating speed and, thus, film thickness had minimal impact on the activity of the films. Instead, the silver doping level and surface roughness played the most significant roles in determining the photocatalytic performance. Higher silver content or multiple photocatalytic coatings increased the surface roughness, but when roughness exceeded an *R*_q_ of 3 µm, the activity decreased. This suggests that while increased roughness can provide a larger surface area, it can also introduce surface defects, reduce the accessibility of active sites, and result in other negative effects that hinder the efficiency of TiO_2_ films. Overall, our findings highlight that optimizing the surface roughness and incorporating silver as a dopant, rather than altering film thickness, is the most effective strategy for creating practical self-cleaning coatings.

## Conflicts of interest

There are no conflicts to declare.

## Supplementary Material

QI-013-D6QI01631F-s001

## Data Availability

The dataset supporting the findings of this study is available on Figshare at https://doi.org/10.6084/m9.figshare.31293928. Supplementary information (SI) is available. See DOI: https://doi.org/10.1039/d6qi01631f.

## References

[cit1] Taylor M., Pullar R. C., Parkin I. P., Piccirillo C. (2020). Nanostructured titanium dioxide coatings prepared by aerosol assisted chemical vapour deposition (AACVD). J. Photochem. Photobiol., A.

[cit2] Sathasivam S., Bhachu D. S., Lu Y., Chadwick N., Althabaiti S. A., Alyoubi A. O., Basahel S. N., Carmalt C. J., Parkin I. P. (2015). Tungsten doped TiO_2_ with enhanced photocatalytic and optoelectrical properties via aerosol assisted chemical vapor deposition. Sci. Rep..

[cit3] Jiamprasertboon A., Kafizas A., Sachs M., Ling M., Alotaibi A. M., Lu Y., Siritanon T., Parkin I. P., Carmalt C. J. (2019). Heterojunction α-Fe_2_O_3_/ZnO films with enhanced photocatalytic properties grown by aerosol-assisted chemical vapour deposition. Chem. – Eur. J..

[cit4] Alotaibi A. M., Promdet P., Hwang G. B., Li J., Nair S. P., Sathasivam S., Kafizas A., Carmalt C. J., Parkin I. P. (2021). Zn and N codoped TiO_2_ thin films: photocatalytic and bactericidal activity. ACS Appl. Mater. Interfaces.

[cit5] Kafizas A., Parry S. A., Chadwick A. V., Carmalt C. J., Parkin I. P. (2013). An EXAFS study on the photo-assisted growth of silver nanoparticles on titanium dioxide thin-films and the identification of their photochromic states. Phys. Chem. Chem. Phys..

[cit6] Pant B., Park M., Park S. J. (2019). Recent advances in TiO_2_ films prepared by sol-gel methods for photocatalytic degradation of organic pollutants and antibacterial activities. Coatings.

[cit7] Dundar I., Krichevskaya M., Katerski A., Krunks M., Acik I. O. (2019). Photocatalytic degradation of different VOCs in the gas-phase over TiO_2_ thin films prepared by ultrasonic spray pyrolysis. Catalysts.

[cit8] Qin Y., Wang Z., Jiang J., Xing L., Wu K. (2020). One-step fabrication of TiO_2_/Ti foil annular photoreactor for photocatalytic degradation of formaldehyde. Chem. Eng. J..

[cit9] Court-Wallace C., Davies P. R., Davies-Jones J., Ososki G. (2023). PiFM and XPS Studies of Porous TiO_2_ Films for the Photocatalytic Decomposition of Polystyrene. Catalysts.

[cit10] Parkin I. P., Palgrave R. G. (2005). Self-cleaning coatings. J. Mater. Chem..

[cit11] Huong H. T., Nhu T. T. Q., Nang H. X., Tuan P. A., Huy P. T. (2023). Super-hydrophilic Ce^+3^-doped TiO_2_-SiO_2_ nanocomposite thin films with high optical transmittance by a simple spin-coating method. Thin Solid Films.

[cit12] Etacheri V., Di Valentin C., Schneider J., Bahnemann D., Pillai S. C. (2015). Visible-light activation of TiO_2_ photocatalysts: Advances in theory and experiments. J. Photochem. Photobiol., C.

[cit13] Varshney G., Kanel S. R., Kempisty D. M., Varshney V., Agrawal A., Sahle-Demessie E., Varma R. S., Nadagouda M. N. (2016). Nanoscale TiO_2_ films and their application in remediation of organic pollutants. Coord. Chem. Rev..

[cit14] Pant B., Park M., Park S. J. (2020). Hydrothermal synthesis of Ag_2_CO_3_-TiO_2_ loaded reduced graphene oxide nanocomposites with highly efficient photocatalytic activity. Chem. Eng. Commun..

[cit15] Jiang M., Nozaki K., Mokudai T., Nakano Y., Uo M., Yamashita K., Ohara S., Wakabayashi N. (2025). Enhancing the photocatalytic activity and antibacterial efficiency of TiO_2_ nanosheets via doping with Ag, Cu, or Ce. ACS Appl. Nano Mater..

[cit16] Tee S. Y., Kong J., Koh J. J., Teng C. P., Wang X., Wang X., Teo S. L., Thitsartarn W., Han M. Y., Seh Z. W. (2024). Structurally and surficially activated TiO_2_ nanomaterials for photochemical reactions. Nanoscale.

[cit17] Tinoco Navarro L. K., Jaroslav C. (2023). Enhancing photocatalytic properties of TiO_2_ photocatalyst and heterojunctions: a comprehensive review of the impact of biphasic systems in aerogels and xerogels synthesis, methods, and mechanisms for environmental applications. Gels.

[cit18] AlMohamadi H., Awad S. A., Sharma A. K., Fayzullaev N., Távara-Aponte A., Chiguala-Contreras L., Amari A., Rodriguez-Benites C., Tahoon M. A., Esmaeili H. (2024). Photocatalytic Activity of Metal- and Non-Metal-Anchored ZnO and TiO_2_ Nanocatalysts for Advanced Photocatalysis: Comparative Study. Catalysts.

[cit19] Singh J., Sahu K., Pandey A., Kumar M., Ghosh T., Satpati B., Som T., Varma S., Avasthi D. K., Mohapatra S. (2017). Atom beam sputtered Ag-TiO_2_ plasmonic nanocomposite thin films for photocatalytic applications. Appl. Surf. Sci..

[cit20] Wu M. C., Lin T. H., Hsu K. H., Hsu J. F. (2019). Photo-induced disinfection property and photocatalytic activity based on the synergistic catalytic technique of Ag doped TiO_2_ nanofibers. Appl. Surf. Sci..

[cit21] Yuan X., Liang S., Ke H., Wei Q., Huang Z., Chen D. (2020). Photocatalytic property of polyester fabrics coated with Ag/TiO_2_ composite films by magnetron sputtering. Vacuum.

[cit22] Li X., Sun N., Bai Y., Yan Y., Ouyang T., Wang X., Jiang X., Wang Z., Cai X., Cai J., Tan H. (2023). High photocatalytic hydrogen production of Ag@TiO_2_ with different sizes by simple chemical synthesis. Langmuir.

[cit23] Li X., Xu H., Ku C., Ye X., Chen J., Feng G., Liu Z., Yang W. (2025). Photocatalytic decarboxylative alkenylation of fatty acids for coproduction of linear α-olefin and hydrogen with high selectivity. ACS Catal..

[cit24] Demirci S., Dikici T., Yurddaskal M., Gultekin S., Toparli M., Celik E. (2016). Synthesis and characterization of Ag doped TiO_2_ heterojunction films and their photocatalytic performances. Appl. Surf. Sci..

[cit25] Gogoi D., Namdeo A., Golder A. K., Peela N. R. (2020). Ag-doped TiO_2_ photocatalysts with effective charge transfer for highly efficient hydrogen production through water splitting. Int. J. Hydrogen Energy.

[cit26] Bensouici F., Souier T., Dakhel A. A., Iratni A., Tala-Ighil R., Bououdina M. (2015). Synthesis, characterization and photocatalytic behavior of Ag doped TiO_2_ thin film. Superlattices Microstruct..

[cit27] Chakhtouna H., Benzeid H., Zari N., el kacem Qaiss A., Bouhfid R. (2021). Recent progress on Ag/TiO_2_ photocatalysts: photocatalytic and bactericidal behaviors. Environ. Sci. Pollut. Res..

[cit28] Fu C., Liu L., Zhao G. (2024). Role of Ag Loading on the concentration of surface-reaching photoexcited holes in TiO_2_ nanoparticles. J. Phys. Chem. C.

[cit29] Bhardwaj S., Sharma D., Kumari P., Pal B. (2020). Influence of photodeposition time and loading amount of Ag co-catalyst on growth, distribution and photocatalytic properties of Ag@TiO_2_ nanocatalysts. Opt. Mater..

[cit30] Komaraiah D., Radha E., Sivakumar J., Ramana Reddy M. V., Sayanna R. (2020). Photoluminescence and photocatalytic activity of spin coated Ag^+^ doped anatase TiO_2_ thin films. Opt. Mater..

[cit31] Jaafar N. F., Jalil A. A., Triwahyono S., Efendi J., Mukti R. R., Jusoh R., Jusoh N. W. C., Karim A. H., Salleh N. F. M., Suendo V. (2015). Direct in situ activation of Ag^0^ nanoparticles in synthesis of Ag/TiO_2_ and its photoactivity. Appl. Surf. Sci..

[cit32] Mills A., Wang J. (2006). Simultaneous monitoring of the destruction of stearic acid and generation of carbon dioxide by self-cleaning semiconductor photocatalytic films. J. Photochem. Photobiol., A.

[cit33] Casado C., Timmers R., Sergejevs A., Clarke C. T., Allsopp D. W. E., Bowen C. R., van Grieken R., Marugán J. (2017). Design and validation of a LED-based high intensity photocatalytic reactor for quantifying activity measurements. Chem. Eng. J..

[cit34] Sergejevs A., Clarke C. T., Allsopp D. W. E., Marugan J., Jaroenworaluck A., Singhapong W., Manpetch P., Timmers R., Casado C., Bowen C. R. (2017). A calibrated UV-LED based light source for water purification and characterisation of photocatalysis. Photochem. Photobiol. Sci..

[cit35] Morgan D. J. (2025). The utility of adventitious carbon for charge correction: A perspective from a second multiuser facility. Surf. Interface Anal..

[cit36] Chinnabathini V. C., Dingenen F., Borah R., Abbas I., van der Tol J., Zarkua Z., D'Acapito F., Nguyen T. H. T., Lievens P., Grandjean D., Verbruggen S. W., Janssens E. (2023). Gas phase deposition of well-defined bimetallic gold-silver clusters for photocatalytic applications. Nanoscale.

[cit37] Alsidran S. H., Court-Wallace C., Davies P. R., Guan S., Morgan D. J., Ososki G. (2024). The role of Cu and film thickness on the photocatalytic activity of mesoporous spin coated TiO_2_ films. Catal. Today.

[cit38] Šuligoj A., Cerc Korošec R., Žerjav G., Novak Tušar N., Lavrenčič Štangar U. (2022). Solar-driven photocatalytic films: synthesis approaches, factors affecting environmental activity, and characterization features. Top. Curr. Chem..

[cit39] Hosseini Z. S., Haghparast F., Masoudi A. A., Mortezaali A. (2022). Enhanced visible photocatalytic performance of un-doped TiO2 nanoparticles thin films through modifying the substrate surface roughness. Mater. Chem. Phys..

[cit40] Lee S. H., Yamasue E., Okumura H., Ishihara K. N. (2015). Effect of substrate roughness and working pressure on photocatalyst of N-doped TiO_x_ films prepared by reactive sputtering with air. Appl. Surf. Sci..

[cit41] Takeda S., Suzuki S., Odaka H., Hosono H. (2001). Photocatalytic TiO_2_ thin film deposited onto glass by DC magnetron sputtering. Thin Solid Films.

[cit42] Wei Y., Wu Q., Meng H., Zhang Y., Cao C. (2023). Recent advances in photocatalytic self-cleaning performances of TiO_2_-based building materials. RSC Adv..

[cit43] Bahruji H., Bowker M., Davies P. R., Pedrono F. (2011). New insights into the mechanism of photocatalytic reforming on Pd/TiO_2_. Appl. Catal., B.

[cit44] Arifin N. M., Mhd Noor E. E., Mohamad F., Mohamad N. (2024). The impact of spinning speed on n-TiO_2_/ZnO bilayer thin film fabricated through sol–gel spin-coating method. Coatings.

