# Supplementary material for: On the relationship between silver concentration, surface roughness and photocatalytic activity of spin-coated TiO2 films
Source: Inorg Chem Front. 2026 Aug 10;13(18):7638–46. doi: 10.1039/d6qi01631f (PMC13576040; doi:10.1039/d6qi01631f)
Supplement: Supplementary file 1 [file QI-013-D6QI01631F-s001.pdf]

This journal is © the Partner Organisations 2026

Supplementary Information

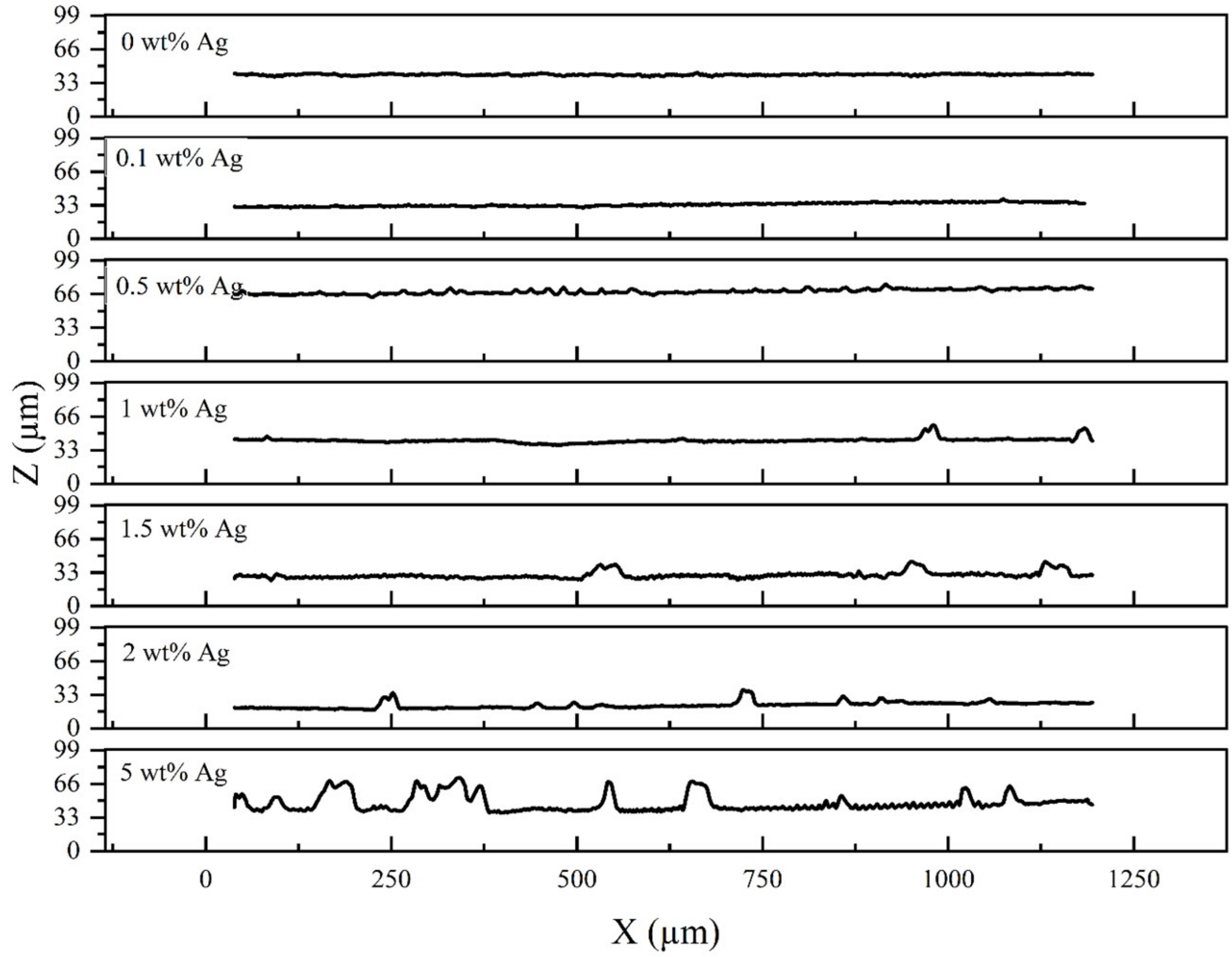

Figure S1. 2D profiles of undoped and 0.1-5 wt% Ag doped-TiO_2 films showing the effect of adding different concentrations of Ag on the TiO_2 surface roughness.

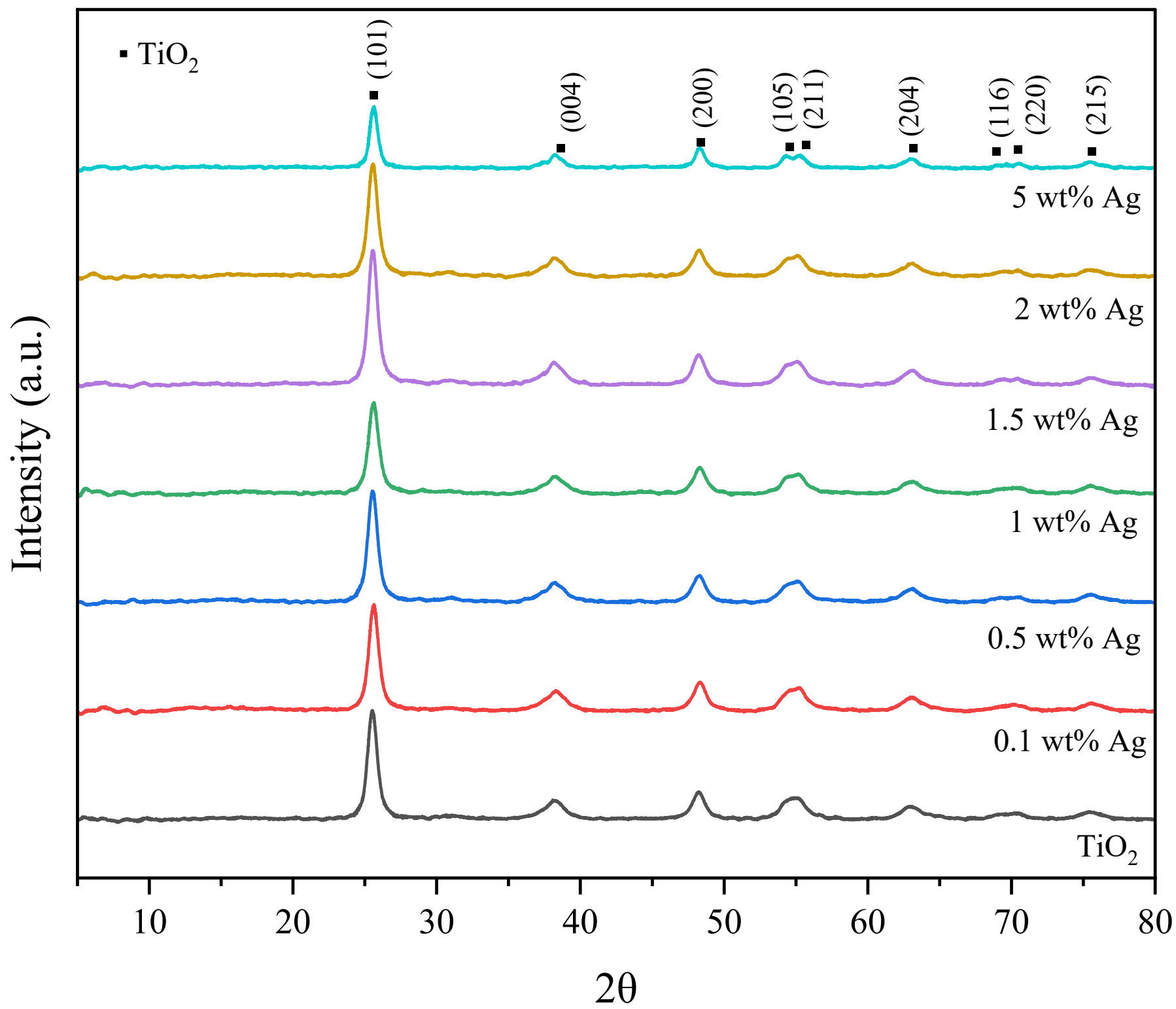

Figure S2. XRD pattern of the pure TiO_2 and silver-doped TiO_2 samples.

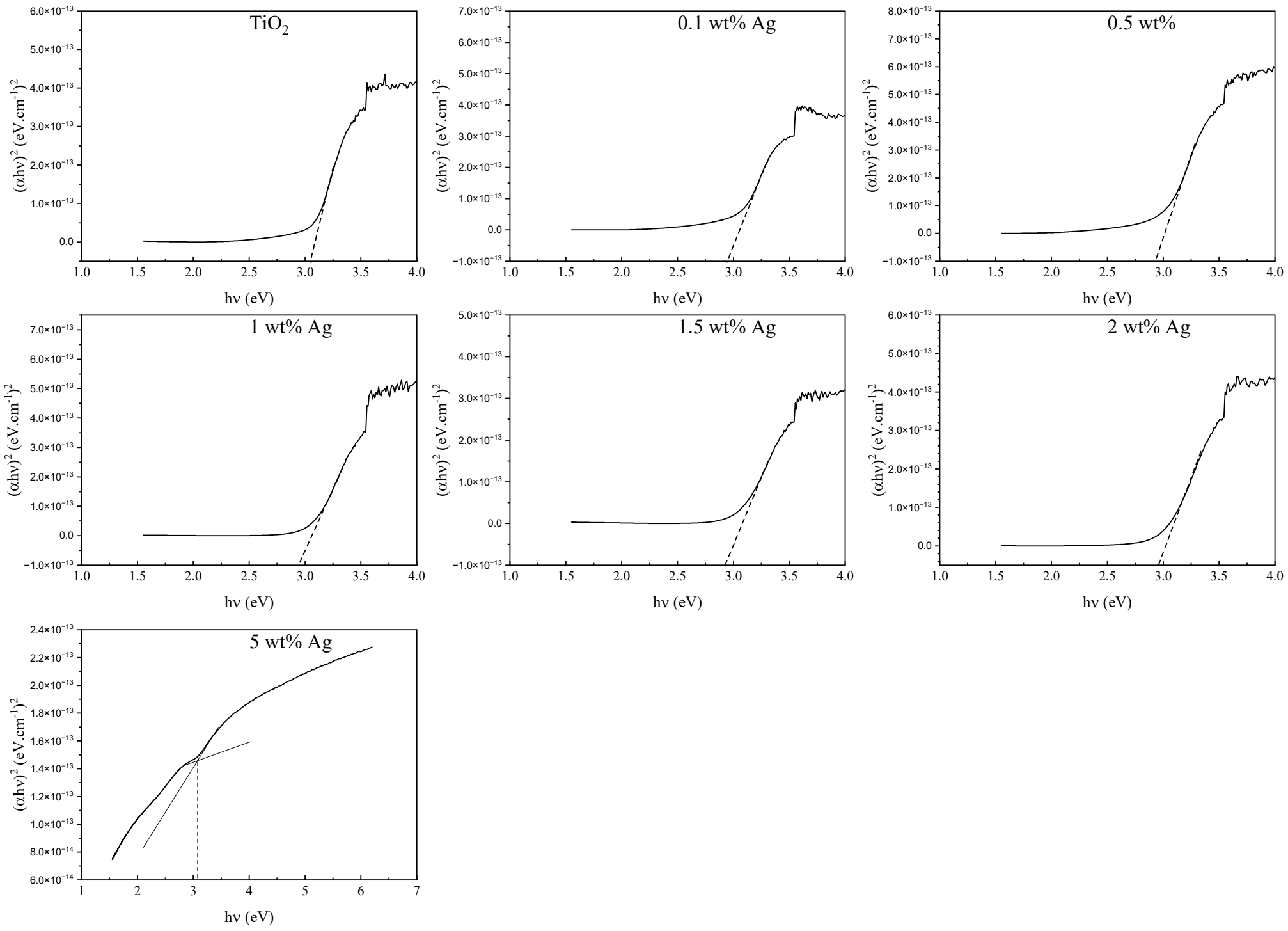

Figure S3. Tauc plots of the pure TiO_2 and silver-doped TiO_2 samples.

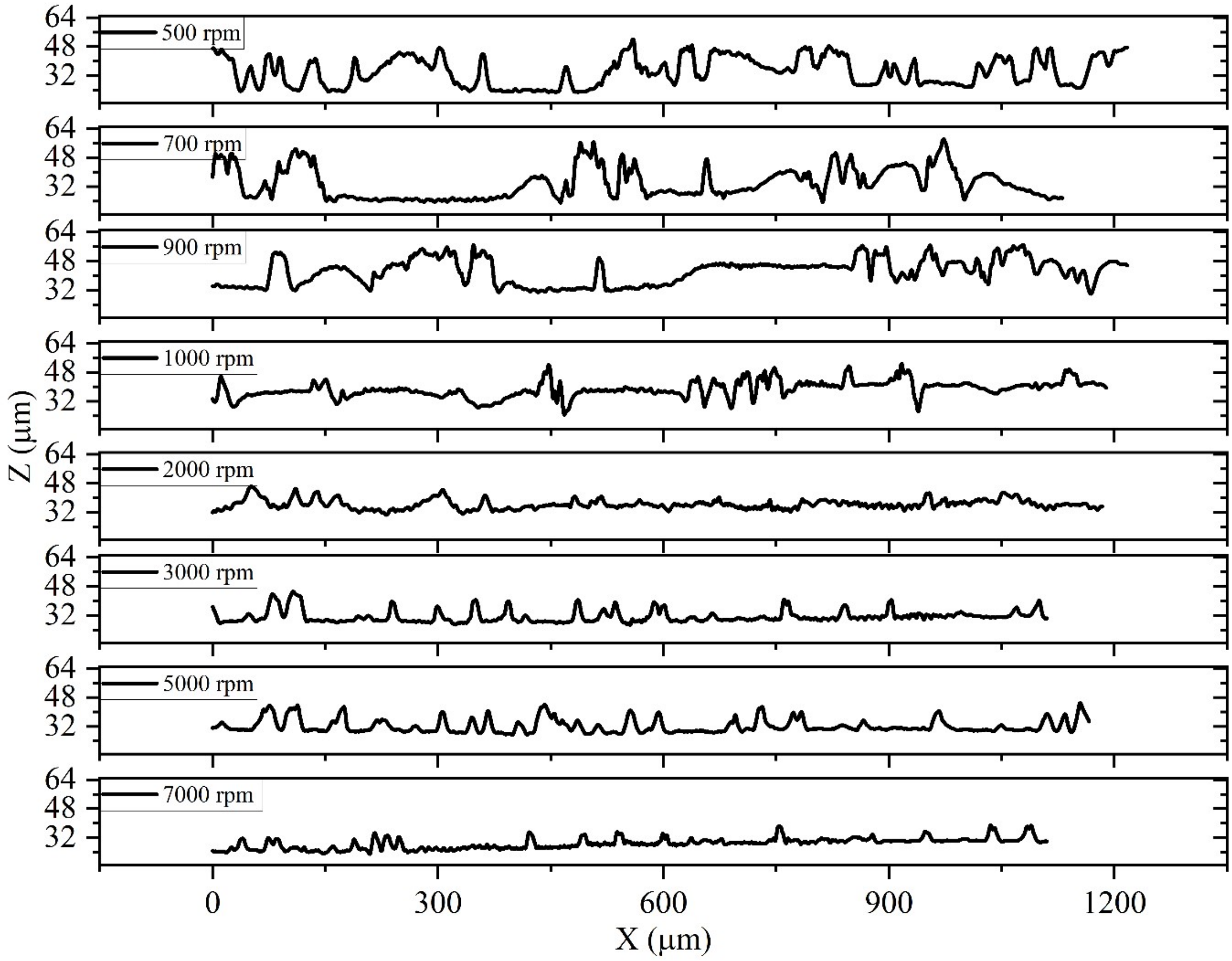

Figure S4. 2D profiles of 1.5 wt% Ag doped-TiO_2 films showing the effect of varying spin coating speed on the surface roughness.

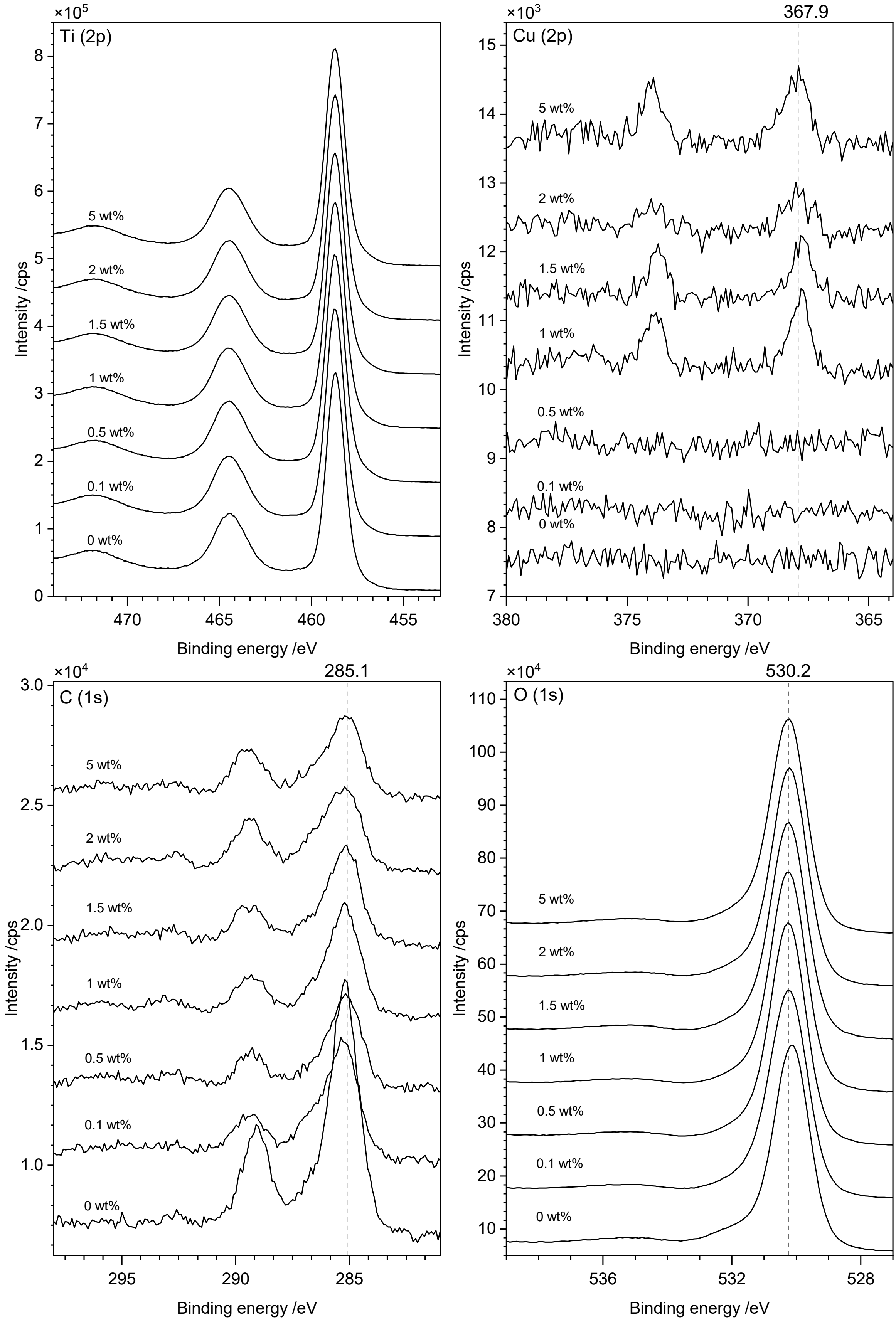

Figure S5. XP spectra of key regions for TiO2 films doped with 0, 0.1, 0.5, 1.0, 1.5, 2.0 and 5.0 wt%

Sample	Water contact angle			
	Fresh		Post UV	
TiO_2	43.2°	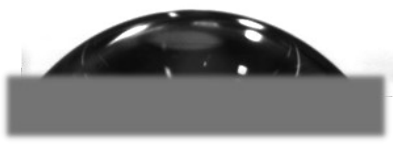	22.2°	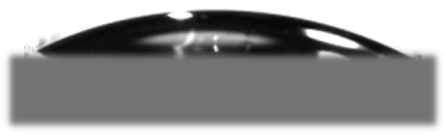
0.1 wt% Ag/TiO_2	39.3°	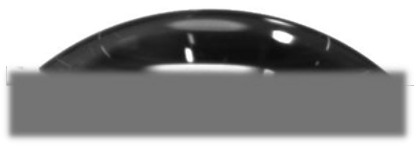	28.2°	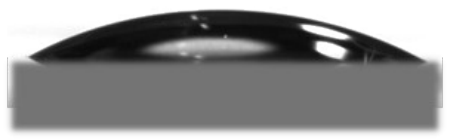
0.5 wt% Ag/TiO_2	52°	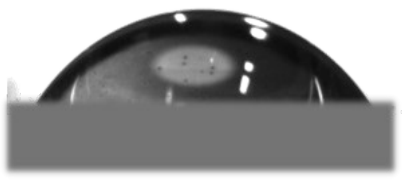	37.3°	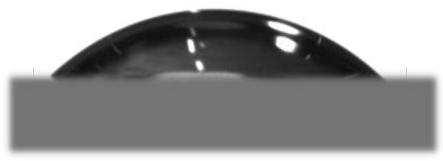
1 wt% Ag/TiO_2	43°	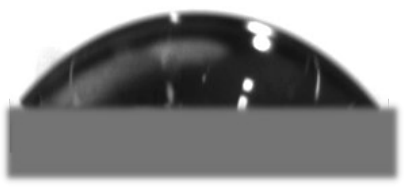	34.6°	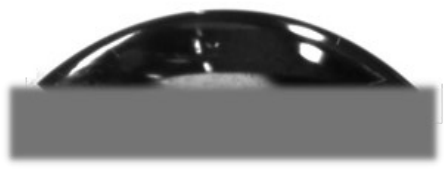
1.5 wt% Ag/TiO_2	39°	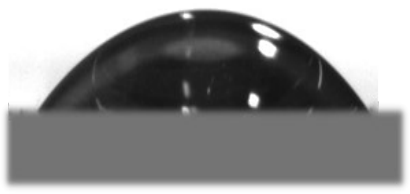	24°	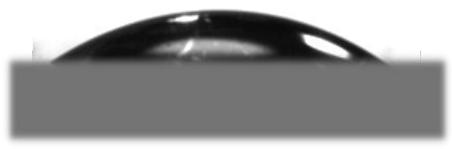
2 wt% Ag/TiO_2	47.3°	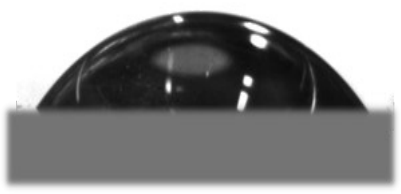	38.5	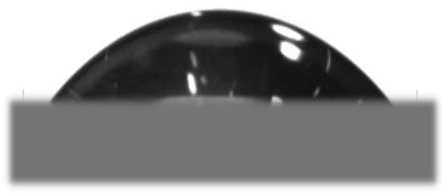
5 wt% Ag/TiO_2	7.4°	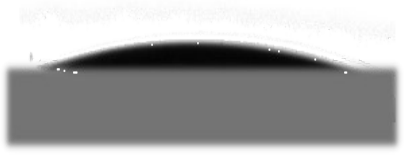	8.3°	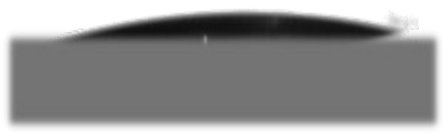

Table S1.Effect of silver concentration and UV treatment on contact angle.
